# Effect of benomyl and diazinon on acquired azole resistance in *Aspergillus flavus* and expression of *mdr1* and *cyp51c* genes

**DOI:** 10.18502/cmm.5.2.1158

**Published:** 2019-06

**Authors:** Maryam Akbari Dana, Seyed Jamal Hashemi, Roshanak Daei Ghazvini, Sadegh Khodavesi, Mona Modiri, Ladan Nazemi, Sima Darabian, Sasan Rezaie

**Affiliations:** 1 Division of Molecular Biology, Department of Medical Mycology and Parasitology, School of Public Health, Tehran University of Medical Sciences, Tehran, Iran; 2 Department of Medical Mycology and Parasitology, School of Public Health, Tehran University of Medical Sciences, Tehran, Iran; 3 Department of Medical Biotechnology, School of Advanced Technologies in Medicine, Tehran University of Medical Sciences, Tehran, Iran; 4 Department of Medical Mycology and Parasitology, School of Public Health, International Campus, Tehran University of Medical Sciences, Tehran, Iran

**Keywords:** Aspergillus flavus, Azole resistant, Gene expression, Pesticides

## Abstract

**Background and Purpose:**

*Aspergillus flavus* is an important pathogen in immunodeficient patients. Due to the abundance of this fungus in nature, fungicides are commonly used to preserve and maintain agricultural products. Long-term exposure to these pesticides can lead to the induction of drug resistance in this fungus.

**Materials and Methods:**

For the purpose of the study, 10 strains of *A. flavus* ATCC 204304 were cultured in benomyl and diazinon pesticides at the concentrations of 62.5, 125, 250.500, 750, 1000, 1500, 2000, and 2500 mg/L in nine steps. Morphological changes and resistance to voriconazole, itraconazole, and amphotericin B were evaluated at the end of each step. Subsequently, changes in the expression of *mdr1* and *cyp51C* genes were studied in the strains showing drug resistance.

**Results:**

The results showed that during the nine stages of the adjacency of strains with benomyl and diazinon at different concentrations, resistance to voriconazole, itraconazole, and amphotericin B in these toxins increased by 30% and 10%, respectively. In addition, the microscopic examination of resistant strains revealed the absence of sporulation, and only mycelium was found. Macroscopically, the color of the colonies changed from green to white. Furthermore, the investigation of the expression of *mdr1* and *cyp51c* genes in these strains showed a decrease and increase in adjacency with diazinon and benomyl, respectively.

**Conclusion:**

As the findings indicated, exposure to agricultural pesticides can lead to the incidence of morphological changes and resistance to amphotericin B, itraconazole, and voriconazole in the sensitive species of *A. flavus* by altering the expression of genes involved in drug resistance.

## Introduction

*Aspergillus flavus*, as one of the most common fungal agents widely found in the environment, is one of the most common causes of aspergillosis with a wide clinical manifestation in immunocompromised patients. Azole drugs (e.g., voriconazole and itraconazole) and non-azole drugs (e.g., amphotericin B) are commonly used to treat these infections [1-3]. In recent years, several reports have been published regarding the increase in drug resistance in *A. flavus*. The genetic evaluation of drug resistance has shown that changes in the expression of *cyp51C* and *mdr1* genes are one of the most important mechanisms for drug resistance in these species [4-6].

Given the distribution of *A. flavus* in nature, this species is known as one of the common cause of fungal infections in agricultural products, resulting in the widespread use of fungicides [7-9]. Benomyl and diazinon are pesticides which are widely used to prevent and manage fungal diseases in agricultural products. Accordingly, the long exposure of these fungi to the mentioned fungicides can be considered as a factor inducing drug resistance in these species [9-12].

Regarding this, the aim of the present study was to evaluate the effect of antifungal benomyl and diazinon pesticides on the morphological characteristics of *A. flavus*. In addition, this study was targeted toward investigating the role of these pesticides in the induction of antifungal resistance in* A. flavus* and expression of *cyp51C* and *mdr1* genes in this species.

## Materials and Methods

***Research samples***

This research was conducted on 10 wild-type strains of *A. flavus* ATCC 204304. 

***Antifungal susceptibility test***

At first, the strains were cultured on a potato dextrose agar (PDA; Merck, Germany) medium. Then, minimum inhibitory concentration (MIC) was evaluated for each strain by the broth microdilution technique, according to the CLSI M38-A2 method [13]. Voriconazole (Pfizer, NY, USA), itraconazole (Janssen Research Foundation, Beerse, Belgium), and amphotericin B (Bristol-Myers-Squibb, Woerden, the Netherlands ) were obtained as reagent-grade powders dissolved in dimethyl sulfoxide, and then diluted in a standard RPMI 1640 medium (Sigma-Aldrich, St. Louis, MO, USA) buffered at a pH of 7.0 with 0.165 mol.L^-1^ morpholine propanesulfonic acid buffer with L-glutamine without bicarbonate (MOPS, Sigma-Aldrich, St. Louis, MO, USA). 

Fungal suspensions were prepared from cultures, using sterile distilled water. The optical density of the supernatant was adjusted spectrophotometrically at 530 nm. The final concentrations of the stock inoculum suspensions were within the range of 0.5-4×10^4^ CFU/ml. In the next stage, serial dilutions (i.e., 0.0313, 0.0625, 0.125, 0.25, 0.5, 1, 2, 4, 8, and 16 μg/ml) of the above antifungal drugs were prepared in a 96-well micro-dilution plate, and fungal suspensions were added to each well. Microplates were stored at 32-35°C for 48 h. *Candida paraposilosis* strains (ATCC22019) and *C. Cruzei* (ATCC6258) were used for quality control.

***Induction of resistance***

In order to investigate the effect of the anti-fungal agent on the outbreak of resistance in the susceptible strains, an in vitro induction assay was performed for a period of 27 weeks using benomyl and diazinon pesticides [14]. To this end, the samples were cultured in a medium of Sabouraud dextrose agar (SDA) at 35°C for 48 h. Fungal suspension was prepared at a concentration of 5×10^4^ CFU/ml in 0.025% normal saline containing 0.85% Tween 20. Subsequently, the serial dilutions of benomyl (China Cytochem) and diazinon (Shimiagro Company) were prepared at the dilutions of 62.5, 125, 250, 500, 750, 1000, 1500, 2000, and 2500 mg/l using sterile distilled water. 

In the first step, 20 μl of the suspensions were added to the SDA medium containing different concentrations of diluted pesticides and kept at 35°C for 3 weeks. At the end of the 3 weeks, MIC and morphological changes, including the color and texture features of the colony (i.e., flat, granular, downy to powdery, and radial grooves), were investigated, and the plate showing the MIC was used to continue the study. 

In the second step, the new suspension was propagated to the plates with a higher dilution of the pesticides. After 3 weeks, the morphological characteristics and MIC values of the samples were examined under standard conditions. The sample with MIC was used to perform the next stage. This procedure was repeated serially for 27 weeks until fungal growth showed resistance to itraconazole, voriconazole, and amphotericin B.

***Study of gene expression***

In the next step, the expressions of *cyp51C* and *mdr1* genes were studied in the samples that showed antifungal resistance using the real-time polymerase chain reaction (RT-PCR). To this end, the RNA samples were extracted using the RNX-PLUS kit (SINACLON). Then, their genomic DNAs were removed by means of the DNase kit (Fermentas, USA). Complementary DNA was synthesized using the PrimeScript RT kit (Fermentase, USA). The changes in the expression of *cyp51C* and *mdr1* genes were investigated by means of the primers designed in Gene Ranger software. In addition, the beta-actin gene was used as the housekeeping gene (Table 1).

***Statistical analysis***

All data analyses were performed in SPSS software for Windows (version 16), using the t-test and Fisher’s exact test. A p-value less than 0.05 was considered statistically significant. The REST® software was used to analyze the RT-PCR data.

**Table 1:** Primers used to investigate the expression of *mdr1* and *cyp51c* genes

***cyp51C***	SENSE	5'-CATGGCCCTGAATGTCACCT-3'
	ANTI- SENSE	5'-GATGAATTCGTTGCCCTGCG-3'
***mdr1***	SENSE	5'-GGTGCTGGGGAGATCACAAC-3'
	ANTI- SENSE	5'-TTCCAGTTCTTTATATAGGCGATGATG-3'
**ßActin**	SENSE	5'-ACgg TAT TTCCA ACTgggACg-3'
	ANTI- SENSE	5'-TggAgCTTCggTCAACAAAACTgg-3'

## Results

**Figure 1:**
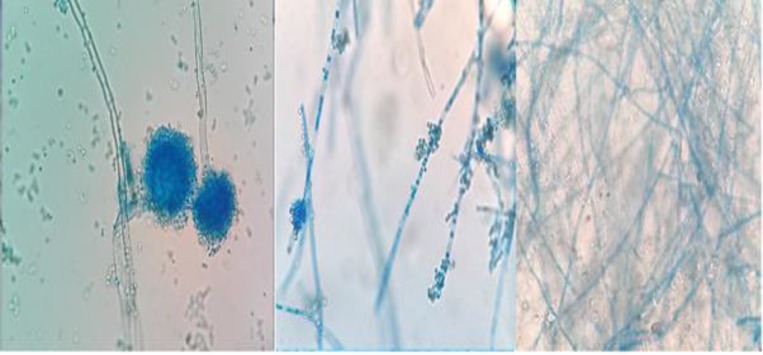
Morphology of *Aspergillus flavus* exposed to benomyl; from left to right: 1) standard sample in the absence of pesticide, 2) stage 4 of the exposure of sample 9 to pesticide, and 3) stage 8 of the exposure of sample 9 to pesticide

The standard samples of *A. flavus* were subjected to antifungal susceptibility testing. The results were indicative of the susceptibility of the samples to voriconazole, itraconazole, and amphotericin B (MIC<2) according to the CLSI guidelines. After the adjacency of the samples with the pesticides of diazinon and benomyl, three strains (i.e., Nos. 4, 6, and 9) had morphological changes among the strains that were exposed to benomyl pesticide. In addition, the colony color of these strains was changed from green to white (Figure 1). Furthermore, these strains showed resistance to antifungal agents (Table 2). 

**Table 2:** Study of the effect of benomyl on the drug resistance after 9 stage

**Exposure to benomyl (after nine stages)**										**Standard strain (without pesticide)**	**Resistance CLSI guideline (µg/ml)**	
**Strain** **10**	**Strain** **9**	**Strain** **8**	**Strain** **7**	**Strain** **6**	**Strain** **5**	**Strain** **4**	**Strain** **3**	**Strain** **2**	**Strain 1**			
<2	8	<2	<2	8	<2	8	<2	<2	<2	<2	<2	**AMB**
0.5	16	0.5	0.5	8	0.5	8	1	0.5	0.5	0.5	<2	**ITC**
1	8	1	<2	8	1	4	<2	1	1	1	<2	**VRC**

AMB: amphotericin B, ITC: itraconazole, VRC: voriconazole

**Table 3:** Study of the effect of diazinon on drug resistance after nine stages

**Exposure to diazinon (after nine stages)**										**Standard** **strain (without pesticide)**	**Resistance CLSI guideline (µg/ml)**	
**Strain** **10**	**Strain** **9**	**Strain** **8**	**Strain** **7**	**Strain** **6**	**Strain** **5**	**Strain** **4**	**Strain** **3**	**Strain** **2**	**Strain** **1**			
2	<2	2	8	<2	2	<2	<2	<2	2	2	<2	**AMB**
0.5	0.5	1	8	0.5	0.5	0.5	2	0.5	0.5	0.5	<2	**ITC**
1	1	1	4	1	1	1	1	1	1	1	<2	**VRC**

AMB: amphotericin B, ITC: itraconazole, VRC: voriconazole

**Figure 2:**
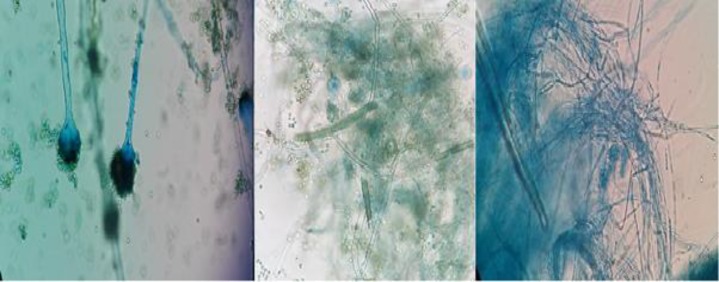
Morphology of *Aspergillus flavus* exposed to diazinon; from left to right: 1) standard sample in the absence of pesticide, 2) stage 5 of the exposure of sample 7 to pesticide, 3) stage 9 of the exposure of sample 7 to pesticide

The occurrence of drug resistance in these strains was seen from the fourth stage onwards. At this stage, the MICs of amphotericin B, itraconazole, and voriconazole were estimated at 8, 4 and 4 μg/ml, respectively. The culture of the samples exposed to diazinon in nine steps revealed morphological changes and drug resistance in one sample (Table 3; Figure 2). The drug resistance in this sample occurred from the fifth stage onwards. At this stage, the MICs of amphotericin B, anthroquanuzole, and voriconazole were 4, 4 and 2 μg/ml, respectively.

**Figure 3:**
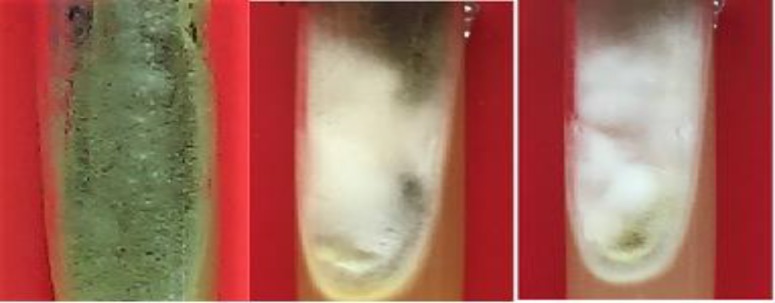
Macroscopic examination of the effect of benomyl on colony formation; left to right: sensitive strain, stage 4, and stage 8

In the macroscopic examination with respect to time and dose elevation of benomyl, macroscopic changes appeared in the culture media that included shallow colonies in white color with smooth and crispy to powdery appearance and reduced growth. However, the major microscopic changes appeared in the exposed fungal cells with short conidiophores, deformed vesicles, and declined conidia (Figure 3).

**Figure 4:**
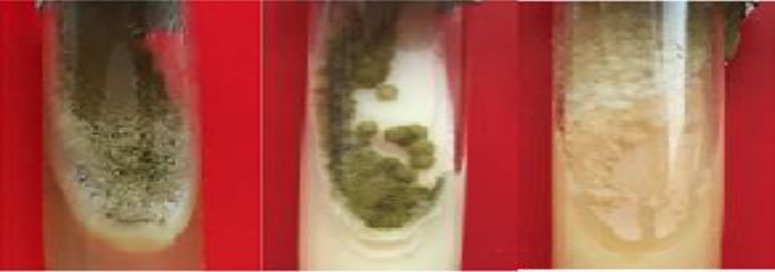
Macroscopic examination of the effect of diazinon on colony formation; left to right: sensitive strain, stage 5, and stage 9

**Figure 5:**
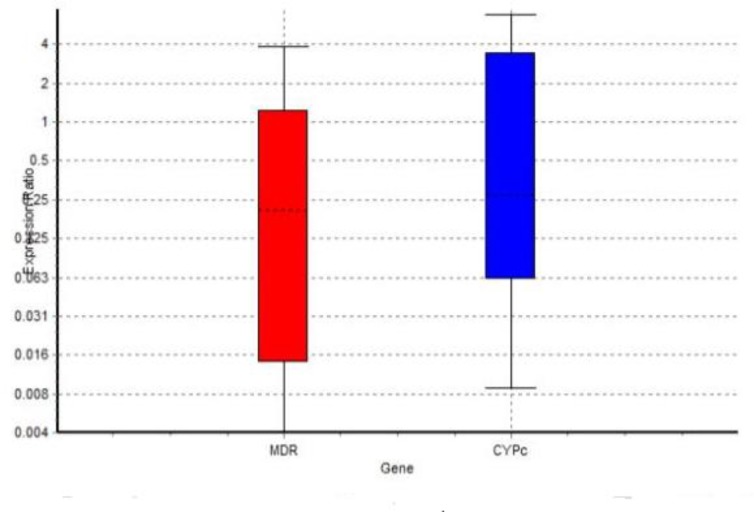
Reduction of gene expression in strain 7 showing drug resistance when exposed to diazinon

In the macroscopic examination of diazinon, a change was observed in the colony color from green to white. The form of colonies changed from a velvet structure to a yeast-like form after they were exposed to pesticides. Furthermore, the sporulation apparatus disappeared, and only mycelium was found (Figure 4). The samples that exhibited higher MICs to antifungal drugs were used to evaluate the gene expression variation via the RT-PCR method. The results showed an increase in the *cyp51c* and *mdr* expression when the samples showed drug resistance in the presence of benomyl pesticide. However, the expression of these genes underwent a decline in sample number 7 (Figure 5). This sample achieved resistance to voriconazole, itraconazole, and amphotericin B when cultured in diazinon pesticide. The results obtained from the expression of genes in the presence of benomyl and diazinon are shown in tables 4 and 5.

**Table 4:** Changes in gene expression at the different stages of the exposure of *Aspergillus flavus* to benomyl

**Result**	**Step 9**	**Step 8**	**Step 7**	**Step 6**	**Step5**	**Step 4**	**Sample no.**	**Standard**	**Gene**
Up	4.4	4.1	3.8	3.4	3.1	2.7	4	1	*cyp51c*
Up	4.4	4.2	3.9	3.6	3.2	2.7	6		
Up	4.5	4.3	3.9	3.5	3.3	2.8	9		
---	1	1	1	1	1	1	Others		
Up	3.9	3.5	3.1	2.6	2.4	2.3	4	1	*mdr*
Up	4.0	3.5	3.2	2.6	2.3	2.1	6		
Up	4.2	3.7	3.4	2.8	2.5	2.2	9		
---	1	1	1	1	1	1	Others		
---	1	1	1	1	1	1		1	Β-actin

The genes expression in samples 4, 6, and 9 (showing drug resistance) increased gradually from step 4 to step 9, while in other samples without drug resistance, the level of gene expression was similar to the standard susceptible standard.

**Table 5:** Changes in gene expression at the different stages of the exposure of *Aspergillus flavus* to diazinon

**result**	**Step 9**	**Step 8**	**Step 7**	**Step 6**	**Step5**	**Step 4**	**Sample no.**	**Standard**	**Gene**
Down	0.5	0.5	0.2	0.1	0.1	0.08	7	1	*cyp51c*
------	1	1	1	1	1	1	Other strains		
Down	0.4	0.3	0.3	0.09	0.07	0.05	7	1	*mdr*
------	1	1	1	1	1	1	Other strains		
---	1	1	1	1	1	1		1	Β-actin

The genes expression in sample no. 7 (showing drug resistance) decreased from step 4 to step 9, while in other samples without drug resistance, the level of gene expression was similar to the standard susceptible standard.

## Discussion

Given the increased frequency of the infections caused by *A. flavus* in immunodeficient patients, the incidence of azole resistance in this fungus is one of the most significant clinical challenges [15-17]. Moreover, this fungus is the most important pathogen contaminating the hospital environment and agricultural products. Considering the high value of agricultural products for humans, fungicides are widely used to prevent and treat the fungal contamination of these products. The long exposure of the environmental fungi to these pesticides may induce cross-resistance in these agents. 

Various studies have been carried out to investigate the effects of fungicides and the onset of drug resistance in fungal species [18-21]. In this regard, Snelders (2009) examined the effect of azole pesticides on drug resistance in *Aspergillus* species. The results of the mentioned study demonstrated that the susceptible species of fungi exhibited resistance to antifungal drugs when exposed to fungicides for a long time [22].

In a study conducted by Escribano et al. (2011), azole resistance was evaluated in *A. fumigatus* isolates after prolonged exposure to itraconazole. They reported that these isolates became resistant to itraconazole (MIC>16 g/ml) and posaconazole (MIC>16 g/ml) only after using concentrated inoculum [23]. In another study carried out by Faria-Ramos (2014), the effect of prochloraz was investigated on cross-resistance in *A. fumigatus* isolates. Their results demonstrated that prochloraz exposure resulted in the induction of morphological changes in *A. fumigatus*, evident elevation of MIC value, and development of cross-resistance to posaconazole, itraconazole, and voriconazole [24].

In another study, Tavares (2014) evaluated the antifungal effect of pesticides on azole resistance in *C. glabrata*. In the mentioned study, despite the susceptibility of the early strains of this fungus to common azole drugs, some strains showed high resistance to fluconazole, voriconazole, and posaconazole after the long-term administration of these agents [25].

In this study, due to the large distribution of *A. flavus* in nature, as well as increased drug resistance in fungal species isolated from patients, the probable effect of the fungicides under investigation (i.e., benomyl and diazinon) on inducing drug resistance in the sensitive species of this fungus was evaluated. The results revealed that although all of the early strains were susceptible to conventional antifungal drugs, 30% and 10% of the samples showed antifungal drug resistance after 9 weeks of exposure to benomyl and diazinon, respectively. The occurrence of drug resistance in the samples of this study is indicative of the emergence of cross-resistance in the fungal strains adjacent to common fungicides.

Comparison of gene expression variation in the primary samples with that in the resistant samples showed that regarding the samples highly resistant to antifungal drugs, the level of gene expression increased in the strains that acquired antifungal resistance through exposure to benomyl. On the other hand, in the strains exposed to diazinon and showed drug resistance, the level of gene expression underwent a decline.

## Conclusion

There are several reports regarding the emergence of resistance to antifungal drugs in patients with *A. flavus* infections. As the results of this study indicated, the long-term exposure of this fungus to fungicides can trigger and induce drug resistance by altering the expression of its genes. Consequently, the use of alternative pesticides can be a good approach to prevent increased drug resistance in this fungus.
